# PKCα phosphorylation of GLT-1 at Ser562/563 induces glutamate excitotoxicity in ischemia in mice

**DOI:** 10.1038/s41392-022-00897-0

**Published:** 2022-03-23

**Authors:** Yuqing Wang, Jun Du, Shanshan Lu, Xia Li, Yifei Chen, Chao Yuan, Sheng-Tao Hou, Yizheng Wang

**Affiliations:** 1 The Brain Science Center, Beijing Institute of Basic Medical Sciences, 100850 Beijing, China; 2 Institute of Neuroscience, Chinese Academy of Sciences, 200031 Shanghai, China; 3 Brain Research Center and Department of Biology, Southern University of Science and Technology, 1088 Xueyuan Blvd, Nanshan District, Shenzhen, 518055 Guangdong Province, China; 4 Huashan Hospital, Fudan University, Shanghai, China

**Keywords:** Diseases of the nervous system, Cell biology

**Dear Editor**,

Glutamate excitotoxicity due to its accumulation in the extracellular space is a major factor to the brain damage that occurs during the early stages of cerebral ischemia^1^. GLT-1 is mainly expressed in astrocytes, and it is responsible for almost 90% of glutamate uptake in the brain^2^. Although GLT-1 upregulation under the administration of ceftriaxone reduces ischemic brain damage, translational application of ceftriaxone in acute ischemia treatment is limited because several days are needed for the upregulation of GLT-1^3^, which misses the critical time window during which suppression of excitotoxicity will be effective.

Our recent work showed that quick modulation of GLT-1 activity by sonic hedgehog (SHH) signaling played a key role in acute cerebral ischemia, and the underlying mechanism included PKCα activation and the phosphorylation of Ser562 (mouse)/563 (rat) on the C terminal of GLT-1^4^. However, it remains unclear whether PKCα interacts with GLT-1, whether SHH regulates this interaction, and what roles they might play during cerebral ischemia. In the current study, we provide evidence to show that PKCα binds and phosphorylates the C terminal of GLT-1 and activation of the SHH pathway increases the interaction between GLT-1 and PKCα. Furthermore, specific disruption of the interaction between PKCα and GLT-1 by peptide or drug alleviates glutamate excitotoxicity and ischemic brain damage.

To determine whether PKCα could interact with GLT-1, we performed mass spectrum analysis on the immunocomplexes from mouse brain homogenate precipitated by the GLT-1 antibody. As shown in Fig. 1a and Supplementary Table S1, GLT-1 and PKCα were found in the same complex. We then observed that PKCα could bind with GLT-1 in GST-pull-down assay (Fig. 1b and Supplementary Fig. 1a, b). These data indicated that PKCα could bind to GLT-1.

**Fig. 1:**
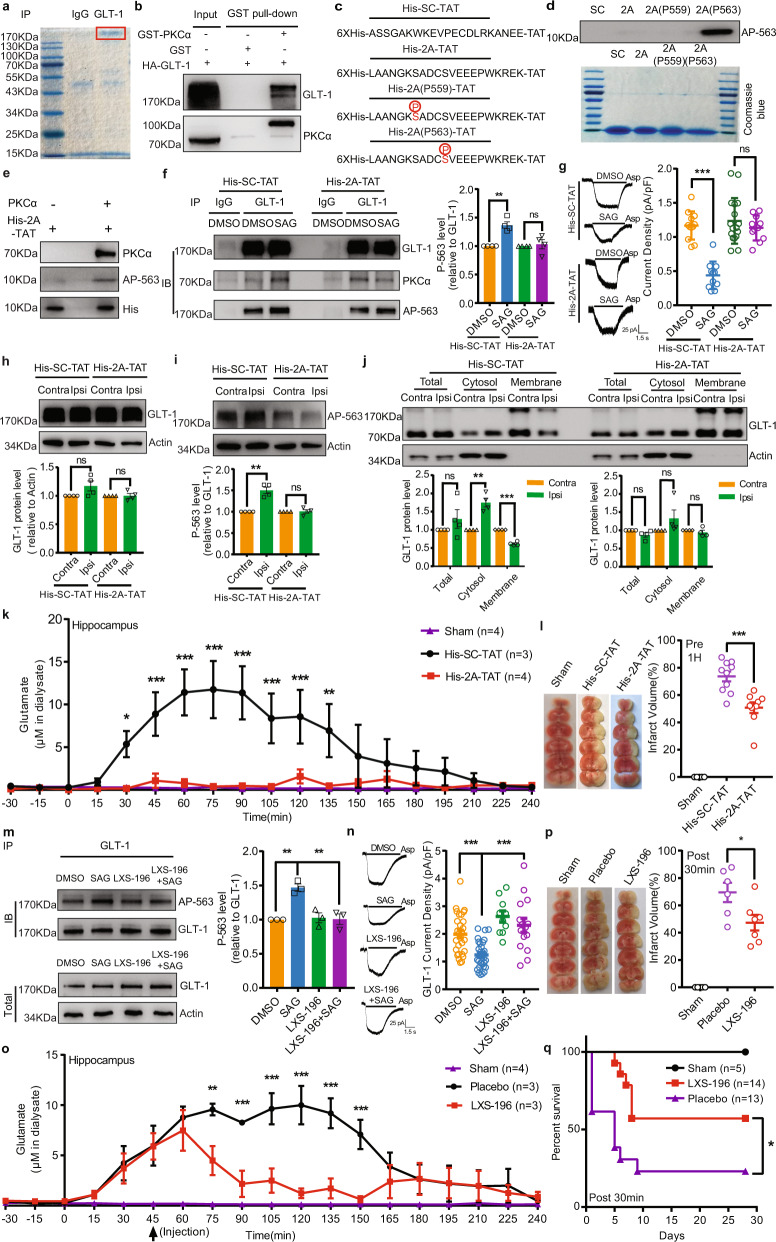
PKCα binds to GLT-1 and phosphorylates it on Ser563 to inhibit its activities during ischemia. **a** Representative Coomassie blue staining image of the immunoprecipitated proteins from mice brain homogenate by GLT-1 antibody. The rectangle indicated the band used for mass spectrum analysis. The mass spectrum result was summarized in Supplementary Table S1, GLT-1 and PKCα were highlighted in red color. **b** Representative immunoblots of the precipitates by GST pull down to show the interaction between PKCα and GLT-1, bait protein: GST- PKCα, prey protein: HA-GLT-1. **c** A diagram showing the sequence of the indicated peptides. Phosphorylated serine was indicated by red color. **d** Upper: representative immunoblots of the four peptides indicated in the figure incubated with AP-563. Lower: representative Coomassie blue staining image of the four peptides. **e** Representative immunoblots of the 1 h in vitro phosphorylation system with the indicated antibodies. **f** Left: representative immunoblots of the immunoprecipitate from astrocytes incubated with His-SC-TAT or His-2A-TAT by the indicated antibodies. Right: statistics, *n* = 4. **g** Aspartate (Asp)-evoked GLT-1 currents in cultured astrocytes incubated with the indicated drugs for 30 min, *n* > 10 in each condition. Left, representative traces; Right, statistics. **h**, **i** Upper: representative immunoblots of the hippocampus lysate from contralateral or ipsilateral 30 min after MCAO with the indicated antibodies. Lower: statistics, *n* = 4. **j** Upper: representative immunoblots of the total, cytosol and membrane fractions of hippocampus taken from mice suffered for 1 h MCAO with His-SC-TAT or His-2A-TAT given 1 h before MCAO. Lower: statistics, *n* = 4. **k** Microdialysis and HPLC analysis of extracellular glutamate in the hippocampus during 2 h MCAO and 2 h reperfusion. His-SC-TAT or His-2A-TAT was given 1 h before MCAO, *n* = 4 in Sham group, *n* = 3 in His-SC-TAT group and *n* = 4 in His-2A-TAT group. **l** Left: representative TTC staining of brain slices from mice subjected to 2 h MCAO and 24 h reperfusion. His-SC-TAT or His-2A-TAT was given 1 h before MCAO. Right: statistics, *n* = 5 in sham group, *n* = 10 in His-SC-TAT group and *n* = 9 in His-2A-TAT group. **m** Left: representative immunoblots of the immunoprecipitate from astrocytes incubated with the indicated agents by the indicated antibodies. Right: statistics, *n* = 3. **n** Aspartate (Asp)-evoked GLT-1 currents in cultured astrocytes incubated with the indicated drugs for 30 min, *n* > 9 in each condition. Left, representative traces; Right, statistics. **o** Microdialysis and HPLC analysis of extracellular glutamate in the hippocampus during 2 h MCAO and 2 h reperfusion. Placebo or LXS-196 was given 45 min after MCAO, *n* = 4 in Sham group, *n* = 3 in Placebo group and *n* = 3 in LXS-196 group. **p** Left: representative TTC staining of brain slices from mice subjected to 2 h MCAO and 24 h reperfusion. Placebo or LXS-196 was given 30 min after MCAO. Right: statistics, *n* = 5 in sham group, *n* = 6 in placebo group and *n* = 7 in LXS-196 group. **q** Evaluation of survival rate during the following four weeks after MCAO. LXS-196 or placebo was given 30 min after MCAO. *n* = 5 in sham group, *n* = 13 in placebo group and *n* = 14 in LXS-196 group. Data are means ± SEM, ns, no significance. *p < 0.05, **p < 0.01 and ***p < 0.001 by two-tailed Student’s *t* test in (**f**–**j)**, by two-way ANOVA with Bonferroni’s multiple comparisons in (**k**) and (**o**), by one-way ANOVA with Bonferroni’s multiple comparisons in (**l**–**n**) and (**p**) and by log-rank test in (**q**)

Further, we designed four peptides, His-SC-TAT, His-2A-TAT, His-2A(P559)-TAT and His-2A(P563)-TAT (Fig. 1c). The His-2A-TAT contained the last 21 amino acids of GLT-1 and His-SC-TAT was used as the scramble control. The His-2A(P559)-TAT or His-2A(P563)-TAT containing the phosphorylated serine at 559 or 563, respectively, was used to test the specificity of the antibody, AP-563. To directly check the phosphorylation status of Ser562/563 (mouse/rat) on GLT-1, we developed the antibody AP-563 and found that AP-563 could specifically recognize His-2A(P563)-TAT, which suggested that it could be used to detect the phosphorylation of Ser562/563 on GLT-1 (Fig. 1d). Next, we built up the in vitro phosphorylation system, in which His-2A-TAT was incubated with or without purified PKCα. We found that the phosphorylation of Ser562/563 was significantly increased after incubating with PKCα for 1 h and 2 h (Fig. 1e and Supplementary Fig. 1c). These results showed that PKCα could directly phosphorylate Ser562/563 on the C terminal of GLT-1.

Then, we found that SAG, the agonist of SHH pathway, increased the binding between PKCα and GLT-1 in cultured astrocytes (Supplementary Fig. 1d–f). To further explore whether the interaction between PKCα and GLT-1 is important to the modulation of GLT-1 activity induced by SHH signal, we incubated His-2A-TAT, a peptide designed to interrupt the interaction between PKCα and GLT-1, and its control peptide, His-SC-TAT, in cultured astrocytes. These two peptides were detected in the cell lysates, suggesting that they had passed through the membrane (Supplementary Fig. 1g). The increased binding between PKCα and GLT-1 (Supplementary Fig. 2a–d) and the phosphorylation of Ser562/563 on GLT-1 (Fig. 1f and Supplementary Fig. 1h) were both abolished in the His-2A-TAT group but not in the His-SC-TAT group. Then, we observed that the decreased membrane expression of GLT-1 (Supplementary Fig. 2e, f) and reduction of GLT-1 activity (Fig. 1g) induced by SAG were reversed after applying His-2A-TAT peptide. Together, these results indicated that the increased phosphorylation of GLT-1 at Ser562/563 by PKCα mediated SHH quick regulation of GLT-1 activity in astrocytes.

To detect the changes in the phosphorylation of Ser562 on GLT-1 in vivo, we first examined the specificity of AP-563 antibody on the hippocampus samples from wild-type (WT) and GLT-1 (S562A) point-mutation mice subjected to middle cerebral artery occlusion (MCAO) (Supplementary Fig. 3a–c). Next, we found that the phosphorylation of Ser562 on GLT-1 was significantly increased in the ipsilateral hippocampus, a representative penumbra area, in the MCAO model of mice (Fig. 1h, i), and His-2A-TAT completely blocked such an increase. Examination of total, cytosol and membrane fractions isolated from the contralateral and ipsilateral hippocampus showed that GLT-1 expression shifted from the membrane to the cytosol in the ipsilateral hippocampus, and this redistribution was inhibited by His-2A-TAT (Fig. 1j). Further, when His-2A-TAT was injected intravenously 1 h before MCAO, the accumulation of extracellular glutamate in the hippocampus was significantly reduced during ischemia (Fig. 1k). More importantly, the ischemic brain damage was alleviated 24 h after MCAO (Fig. 1l). In contrast, the blood flow in the hippocampus did not change after His-2A-TAT injection (Supplementary Fig. 3d). These data provide the direct evidence that increased phosphorylation of Ser562 on GLT-1 promoted the reduced membrane expression of GLT-1 and eventually contributed to glutamate excitotoxicity in ischemia.

Then, we used LXS-196, a PKCα inhibitor used for clinical trials on uveal melanoma^5^ (Supplementary Fig. 4a). Similar to His-2A-TAT treatment, LXS-196 completely blocked the increased binding between PKCα and GLT-1 following SAG induction in cultured astrocytes (Supplementary Fig. 4b, c). Moreover, LXS-196 reversed the increased phosphorylation of Ser563 on GLT-1 (Fig. 1m) and sequentially reduced the membrane expression of GLT-1 (Supplementary Fig. 4d) by SAG stimulation. Finally, the reduced GLT-1 activity induced by SAG was recovered by the LXS-196 treatment (Fig. 1n). Taken together, these data indicated that LXS-196 could reverse the quick modulation of GLT-1 activity by SHH signaling through disrupting the interactions between PKCα and GLT-1.

To further explore the translational possibility of LXS-196 for treating cerebral ischemia, we intravenously injected LXS-196 and found that LXS-196 inhibited the increased phosphorylation of Ser562 on GLT-1 in the ipsilateral hippocampus (Supplementary Fig. 5a, b). When LXS-196 was administered 45 min after MCAO, the increased phosphorylation of Ser562 on GLT-1 in the ipsilateral hippocampus was reversed (Supplementary Fig. 5c, d), meanwhile, the accumulation of extracellular glutamate in the ipsilateral hippocampus was significantly reduced (Fig. 1o). The blood flow in the hippocampus did not change after the LXS-196 injection (Supplementary Fig. 5e). Then, we intravenously injected either LXS-196 or its solvent 30 min after MCAO and found that both the neurological deficit score and the ischemic brain damage were alleviated in the LXS-196-treated group (Fig. 1p and Supplementary Fig. 5f). Finally, we found that LXS-196 treatment significantly increased mice survival rate during the following four weeks after MCAO (Fig. 1q). These data provided further support to the idea that LXS-196 was promising for clinical translation in treating ischemic stroke.

In summary, the present study identified that modulation of PKCα interaction with GLT-1 by activation of SHH pathway immediately after cerebral ischemia served as a mechanism underlying excitotoxicity in the ischemic brain. Targeting this pathway, such as by using the inhibitor LXS-196 to PKCα, can be developed into an effective clinical therapy to treat ischemic stroke in humans. To be limited, although PKCα directly phosphorylated Ser562/563 on the C terminal of GLT-1 in vitro, it is possible that other proteins or even protein kinases were involved in the regulation of the activities of GLT-1 induced by SHH signal.

## Supplementary information

Supplementary materials

Supplementary table 1-mass spectrum analysis

## Data Availability

Data are available upon reasonable request.
