# Supplementary material for: PKCα phosphorylation of GLT-1 at Ser562/563 induces glutamate excitotoxicity in ischemia in mice
Source: Signal Transduct Target Ther. 2022 Mar 23;7:82. doi: 10.1038/s41392-022-00897-0 (PMC8940927; doi:10.1038/s41392-022-00897-0)
Supplement: Supplementary file 1 — Supplementary materials [file 41392_2022_897_MOESM1_ESM.docx]

Supplementary Materials for

**PKCα phosphorylation of GLT-1 at Ser562/563 induces glutamate excitotoxicity in ischemia in mice**

Yuqing Wang, Jun Du, Shanshan Lu, Xia Li, Yifei Chen, Chao Yuan, Sheng-Tao Hou &Yizheng Wang

Correspondence to: Sheng-Tao Hou (hou.st@sustech.edu.cn) or Yizheng Wang (yzwang@ion.ac.cn).

**This PDF file includes:**

Materials and Methods

Figures. S1 to S5

Table. S1 (attached as a single excel file)

Materials and Methods

**Study approval**

All animal surgeries were strictly followed with the guidelines of animal welfare in Beijing Institute of Basic Medical Sciences, China.

**Plasmids, peptides, reagents and antibodies**

HA-GLT-1a and GST were cloned into pCDH vector. All the peptides were synthesized by Synpeptide. GST protein interaction Pull-Down kit from Pierce (21516), Fast Protein Stain buffer from Biofuraw (180-7001), triphenyl tetrazolium chloride (TDB0558) from Sangon, StarFect High-efficiency Transfection Reagent (C101-10) from GenStar. UCPH101 (3490) from Tocris, LXS-196 (S6723) was purchased from Selleckchem, protease inhibitor cocktail (B14001), protein A/G beads and anti-HA magnetic beads (B26201) from Biotool, Luminata Crescendo Western HRP Substrate (WBLUR0500) and bFGF (GF003) were from Millipore, RIPA lysis buffer (CW2334S) from CWBIO, disuccinimidyl suberate (21655) from Pierce, M270 Streptavidin beads (65306) was from Invitrogen. PKC alpha protein (active, ab55672), Anti-PKC alpha antibody (Y124, 1:1000) from Abcam, anti-GLT-1 antibody (22515-1-AP, 1:1000) from Proteintech, anti-GST antibody (KM8005, 1:1000), anti-His-tag antibody (KM8001, 1:1000) and AP-563 antibody were from Tianjin Sungene Biotech, anti-beta Actin antibody (MA1-140, 1:1000) from Invitrogen, normal rabbit IgG (sc-2027) from Santa Cruz, HRP conjugated goat anti-rabbit antibody (#31460, 1:5000) and HRP-conjugated goat anti-mouse antibody (#31430, 1:5000) from Thermo.

**Mice**

C57 mice were purchased from SPF biotechnology co. LTD. GLT-1 (S562A) mice were confirmed by PCR and sequencing. For peptides and drug injection experiments, mice were randomly assigned into groups as needed. His-SC-TAT or His-2A-TAT (10mg/kg) was injected intravenously 60min before MCAO model. LXS-196 (2mg) was sequentially dissolved by DMSO (100μL), PEG400 (800μL), Tween-80 (100μL) and saline (3mL) for intravenous administration. Each mouse received 6mg/kg LXS-196 injection. The solvent was used as placebo.

**Focal cerebral ischemia, triphenyl tetrazolium chloride (TTC) staining and neurological deficit score in mice**

Mice transient focal cerebral ischemia was induced by middle cerebral artery occlusion (MCAO) and reperfusion. Body temperature was kept at 37℃ during the surgery. At the endpoint of the experiments, the brains were taken out to cut into 2mm slices. The slices were then stained with 1% TTC buffer (dissolved in PBS) at room temperature for 15min. The slices were kept in 4% paraformaldehyde at 4℃. Ischemic brain damage was calculated as described previously^1^, I% = (contralateral volume - healthy ipsilateral volume)/contralateral volume. Neurological deficit was assessed as described previously^2^ in a double-blind fashion: 0, no obvious deficit; 1, torso flexion to the left; 2, spontaneous circling to the left; 3, leaning/falling to the left; 4, no spontaneous movement; 5, death.

**Blood flow measurement**

Mice were first anesthetized and then the detecting probe was implanted into hippocampus (2mm posterior, 1.8mm right and 1.5mm below dura). The value of focal blood flow was shown on the Laser Doppler Perfusion and Temperature Monitor (Moor Instruments) during the MCAO experiments. The body temperature of the mice was kept at 37℃ during the experiments.

**Microdialysis and HPLC analysis**

The CMA/7 guide cannula (P000137, CMA) was implanted into hippocampus (2mm posterior, 1.8mm right and 1.5mm below dura relative to bregma) 7 days before the MCAO model. Before the experiments, mice were anesthetized and CMA 7 microdialysis probe (P000082, CMA) was inserted into the cannula. aCSF (in mM: NaCl 120, KCl 2.7, MgCl_2_ 1.0, CaCl_2_ 1.2, NaHCO_3_ 25mM, ascorbic acid 0.05) was bubbled with 5% CO_2_ and 95% O_2_ for 5min and infused into the microdialysis system at the speed of 1μl/min by Syringe Pump and dialysates were collected every 15min during ischemia. The body temperature of the mice was kept at 37℃ during MCAO and reperfusion. The concentration of glutamate in the dialysates was measured by HPLC analysis.

***In vitro* phosphorylation assay**

His-2A-TAT (1µg) was incubated with 6µl Kinase Assay Buffer III (200mM Tris-HCl, 100mM MgCl_2_ and 0.5mg/ml BSA), 5µl PKC Lipid Activator, 1µl ATP(10mM), 7.5µl DTT（1M）and purified PKCα (0.5µg), ddH_2_O was added to make the *in vitro* phosphorylation system to 30µl. In the control group, PKCα was replaced by ddH_2_O. After incubating at 33°C for 1 hour or 2 hours, the phosphorylation system was stopped by boiling with 4X SDS sample buffer for 10min.

**Primary astrocyte culture, HEK293T cell line and transfection**

Cerebral cortex was isolated from P0 SD rats on ice. After digestion with trypsin for 6min at 37℃, the tissues were filtered to collect brain cells. The brain cells were then seeded in culture flakes with DMEM medium (containing 10% fetal bovin serum) for 9 days. After vibrating for 6-8 hours, the astrocytes on the bottom of the flake were digested and seeded in the six-well plates at the density of 8×10^5^/well with DMEM/F12 medium containing N2 supplement and basic fibroblast growth factor (bFGF, 20 ng/ml) for 3 days before usage. His-SC-TAT or His-2A-TAT (1μM) was incubated with astrocytes for 4h before DMSO or SAG added.

HEK293T cells were cultured with DMEM medium containing 10% fetal bovin serum. 5μg plasmid was mixed with 15μl StarFect High-efficiency Transfection Reagent in 1mL DMEM medium at room temperature for 20min, then the mixture was added into the 10cm dish culture for 48hs before harvest.

**Whole-cell patch-clamp recordings in cultured astrocytes**

Astrocytes were performed whole-cell recordings at room temperature. Patch electrodes were pulled by a Flaming/Brown micropipette puller (P-97, Sutter Instruments). The recording electrodes was filled with internal solutions with resistance of 4–6 MΩ and the liquid junction potential was reset when electrodes touched the external solutions (ES). After formation of whole-cell recording, the access resistances were generally < 20 MΩ.

To record aspartate (100 μM)-evoked currents, the internal solution (in mM: KNO_3_ 140, MgCl_2_ 2.5, EGTA 11, Na_2_ATP 5, and HEPES 10, pH adjusted to 7.3 with KOH) and the ES［in mM: NaCl 135, KCl 5.4, CaCl_2_ 1.8, MgCl_2_ 1.3, glucose 10, HEPES 10, D-CPP-ene (NMDA receptor antagonist) 0.03, CNQX(AMPA/KA receptor antagonist) 0.01, bicuculline (GABA_A_ receptor antagonist) 0.02 and UCPH101 (GLAST inhibitor) 0.01, pH adjusted to 7.4 with NaOH］were used.The membrane potential was held at -80 mV and astrocytes were bathed in ES constantly. Drugs were diluted in ES and applied to astrocytes by pressure using the 8-Channel Focal Perfusion System (ALA Scientific Instruments).

**Immunoprecipitation and mass spectrum analysis**

Mice were anesthetized and sacrificed, then the brain cortex was dissected and lysed in the RIPA (containing protein inhibitor cocktail) buffer at 4℃ for 2h. After centrifugation under 10000g, 4℃ for 15min, the supernatant was transferred to another tube and mixed with anti-GLT-1 antibody at 4℃ overnight. The next day, protein A/G magnetic beads were added and incubated at 4℃ for 3hs. Then the beads were washed with RIPA buffer for 30min at room temperature. Finally, the beads were boiled for 5min in SDS sample buffer to collect the immunoprecipitated proteins. The samples were electrophoresed on SDS-10%PAGE, then the gel was stained in Fast Protein Stain buffer, the corresponding band was cut off and digested with trypsin.

LC-MS/MS analyses were performed on an Easy-nLC 1000 liquid chromatography system (Thermo) coupled to a Q-Exactive HF via a nano-electrospray ion source (Thermo). The peptide mixture was eluted from a 360-um ID x 2cm, C18 trap column and separated on a homemade 150um ID x 15 cm column (C18, 1.9um, 120Å, Dr. Maisch GmbH) with a linear 5-35% acetonitrile gradient at 600 nl/min. Survey scan were acquired after accumulation of 3e6 ions in Orbitrap for m/z 300-1400 using a resolution of 120,000 at m/z 200. The top 30 intense precursor ions were selected for fragmentation in the HCD cell at normalized collision energy of 27%, and then fragment ions were transferred into the Orbitrap analyzer operating at a resolution of 15,000 at m/z 200. The dynamic exclusion of previously acquired precursor ions was enabled at 12 s.

Spectral data were searched against mouse protein RefSeq database in Proteome Discoverer 1.4 .1.14 using Mascot (version 2.3.01, Matrix Science) to achieve a false discovery rate of <1%. The mass tolerance was set to be 20 ppm for precursor, and it was set 50mmu for the tolerance of product ions. Acetyl (Protein-N term), Oxidation (M), DeStreak(C), Phospho(S/T/Y) were chosen as variable modifications. Two missed cleavage sites for trypsin were allowed. Detail data was shown in Supplementary Table S1.

**Co-immunoprecipitation and GST pull-down assays**

For co-immunoprecipitation, cultured astrocytes were rinsed in cold PBS and lysed in the RIPA (containing 1X protein inhibitor cocktail) buffer. After centrifugation under 10000g, 4℃ for 15min, the supernatant was transferred to another tube, then the protein extracts were immunoprecipitated with anti-GLT-1 antibody or normal rabbit IgG at 4℃ overnight. The next day, protein A/G magnetic beads were added and incubated at 4℃ for 3 hours. Then, the beads were washed with RIPA buffer for 30min at room temperature. Finally, the beads were boiled for 5min in SDS sample buffer and the precipitated proteins were separated and detected with standard immunoblot.

GST pull-down assay was conducted as the manufacturers’ protocol. GST gene was cloned into pCDH and transfected HEK293T cells as described above. Cells from two dishes were harvested and lysed 48h later. Bait proteins (the cell lysate or 1μg GST fused PKCα) were immobilized with the glutathione agarose at 4℃ for 3 hours. After washing for five times, the prey protein was incubated with the agarose at 4℃ for 2 hours. Finally, 10mM glutathione elution buffer was added to elute the bait and prey proteins.

**Membrane protein extraction**

Cultured astrocytes were washed with cold PBS twice and incubated with biotin in PBS (1mg/ml) at 4℃ for 25min, then the reaction was stopped by 100mM glycine (in PBS) for 10min at room temperature. The RIPA (containing 1X protein inhibitor cocktail) buffer was used to lyse the astrocytes for 15min on ice. After centrifugation under 10000×g, 4℃ for 15min, the supernatant was transferred into a new tube and mixed with magnetic avidin beads at 4℃ for 2 hours. Then, the beads were washed with cold RIPA buffer for three times. Finally, the beads were boiled in SDS sample buffer to collect the membrane proteins.

The hippocampus was homogenizing in 5×volume of ice-cold homogenizing buffer (Tris-HCl 20mM, pH=7.4, DTT 1mM, EGTA 5mM, EDTA 2mM, glycerol 10%, MgCl_2_ 1mM). After centrifuging under 1,000×g, 4℃ for 30min, the supernatant was collected. Then, the supernatant was centrifuged under 27,000×g, 4℃ for 1 hour, after which the supernatant was picked as the crude cytosolic extract and the pellet was suspended using homogenizing buffer containing 0.5% Triton X-100 at 4℃ for 1h. Finally, the suspended buffer was centrifuged under 27,000×g, 4℃ for 30min and the supernatant was collected as the membrane fraction.

**Western Blot**

The lysates from cells or brain tissues were electrophoresed on SDS-10%PAGE and then transferred to polyvinyldifluoridine membranes (10600023, GE Healthcare Life science). After blocking with 5% milk (dissolved in PBS) at room temperature for 1 hour, the membranes were incubated with the primary antibodies at 4℃ overnight. The membranes were incubated with HRP-conjugated secondary antibodies at room temperature for 2 hours on the next day. Following washing with PBS for 20min, the protein bands were visualized by Tanon 5200 (Tanon) system using Luminata Crescendo Western HRP Substrate. The band density was determined by using ImageJ-2.

**Primers for genotyping and cloning**

GLT-1 (S562A) for genotyping, forward, 5’-CCTAAACTGCTGAGAATCCGT-3’, reverse, 5’-AGCAGCGTCCATTCAAACAA-3’. for cloning GST into pCDH were: sense, 5’-CACGCTAGCGCCACCATGTTGGGTGGTTGTCCAAAA-3’, antisense, 5’-CCCGCTAGCTCAGTCACGATGCGGCC -3’; for cloning HA-GLT-1a into pCDH were: sense, 5’- CACGAATTCATGGCCACC TATCCATATGATGTTCCAGATTATGCAATGGCATCAACCGAGGGTG-3’, antisense, 5’- CCCGAATTCTTATTTTTCACGTTTCCAAGGTTCT-3’.

**Statistical analysis**

Trials and animal numbers used in the experiments were determined by previous experience. Statistical analysis was performed by GraphPad Prism 7 software. Two-tailed Student’s t test was used to compare the differences between two groups, one-way ANOVA with Bonferroni’s multiple comparison was used to compare multiple groups. In Fig 1, Supplementary Fig 3 and Supplementary Fig 5, two-way ANOVA with Bonferroni’s multiple comparison was used to compare the differences. Survival rate was evaluated by log-rank test. The value of p < 0.05 was regarded as statistically significant.

**REFERENCES**

1. Du W. et al. Inhibition of TRPC6 degradation suppresses ischemic brain damage in rats. *J Clin Invest* **120**, 3480-3492 (2010).

2. Li P. et al. Liang, A. W. Thomson, J. Chen, X. Hu, Adoptive regulatory T-cell therapy protects against cerebral ischemia. *Ann Neurol* **74**, 458–471 (2013).

**Figure. S1. Direct interaction between PKCα and GLT-1. a** Representative immunoblots of purified PKCα or total lysate from HEK293T cells overexpression of GST with the indicated antibodies. **b** Representative immunoblots to show the amount of bait proteins in GST-pull down assay. **c** Representative immunoblots of the 2h *in vitro* phosphorylation system with the indicated antibodies. **d** Representative immunoblots of total lysate from cultured astrocytes incubated with the indicated agents for 45min by the indicated antibodies. **e** Left: representative immunoblots of the co-immunoprecipitation assay in astrocytes incubated with the indicated agents for 45min by the indicated antibodies. Right: statistics, n = 4. **f** Representative immunofluorescence image for Hoechst (blue) and GFAP (green) in cultured astrocytes. Scale bar, 100μm. **g** Representative immunoblots of astrocytes lysate with anti-His antibody. **h** Representative immunoblots of total lysate from cultured astrocytes incubated with the indicated agents for 45min by the indicated antibodies. His-SC-TAT or His-2A-TAT was added 4 h before the agents were incubated. Data are means ± SEM. *p < 0.05 by two-tailed Student’s t-test.

**Figure. S2. Inhibition of the interaction between PKCα and GLT-1 reverses the reduction of membrane expression of GLT-1 induced by SAG. a, b** Representative immunoblots of total lysate from cultured astrocytes incubated with the indicated agents for 45min by the indicated antibodies. His-SC-TAT or His-2A-TAT was added 4 h before the agents were incubated. **c, d** Upper: representative immunoblots of the immunoprecipitate from astrocytes incubated with His-SC-TAT or His-2A-TAT by the indicated antibodies. Lower: statistics, n = 4. **e, f** Upper: representative immunoblots of the total lysates or the membrane fractions of cultured astrocytes incubated with the indicated agents for 1h. Lower: statistics, n = 4 in **e** and n = 3 in **f**. Data are means ± SEM, ns, no significance. *p < 0.05 by two-tailed Student’s t-test.

**Figure. S3. Increased phosphorylation of Ser563 on GLT-1 in MCAO. a** Identification of wildtype (WT) and GLT-1 (S562A) mice by sequencing. **b** Representative immunoblots of hippocampus lysate from mice suffered for 30min MCAO with the indicated antibodies. **c** Left: representative immunoblots of the hippocampus lysate from contralateral or ipsilateral 30min after MCAO with the indicated antibodies. Right: statistics, n = 3. **d** Influence of His-SC-TAT and His-2A-TAT on blood flow in the hippocampus during 2h MCAO and 2h reperfusion, n = 3 in each group. Unless stated, CBF, cerebral blood flow. Data are means ± SEM, ns, no significance. Two-tailed Student’s t-test was used in **c**, two-way ANOVA with Bonferroni’s multiple comparisons was used in **d**.

**Figure. S4. LXS-196 reduces the binding between PKCα and GLT-1 induced by SAG. a** The chemical structure of LXS-196, a PKCα inhibitor. The picture was cited from PubChem (CID: 118873253). **b** Representative immunoblots of total lysate from cultured astrocytes incubated with the indicated agents for 45min by the indicated antibodies. **c** Upper: representative immunoblots of the co-immunoprecipitation assay in astrocytes incubated with the indicated agents for 45min by the indicated antibodies. Lower: statistics, n = 3 in each condition. **d** Upper: representative immunoblots of the total lysates or the membrane fractions of cultured astrocytes incubated with the indicated agents. Lower: statistics, n = 3 in each condition. Data are means ± SEM, ns, no significance. *p < 0.05 and **p < 0.01 by one-way ANOVA with Bonferroni’s multiple comparison.

**Figure. S5. LXS-196 reduces the phosphorylation of Ser563 on GLT-1 and builds protective effects in MCAO. a, b** Upper: representative immunoblots of the hippocampus lysate from mice 30min after MCAO with the indicated antibodies. Placebo or LXS-196 was injected 15min before MCAO. Lower: statistics, n = 3. **c, d** Upper: representative immunoblots of the hippocampus lysate from mice 90min after MCAO with the indicated antibodies. Placebo or LXS-196 was injected 45min after MCAO. Lower: statistics, n = 3 in placebo group and n = 4 in LXS-196 group. **e** Influence of placebo and LXS-196 on blood flow in the hippocampus during 2h MCAO and 2h reperfusion, n = 3 in each group. **f** Evaluation of neurological deficit after 24hs reperfusion, placebo or LXS-196 was injected about 30min after MCAO, n = 5 in sham group, n = 19 in the placebo group and n = 21 in LXS-196 group. Data are means ± SEM, ns, no significance. *p < 0.05 and **p < 0.01 by two-tailed Student’s t-test in **a-d**, by two-way ANOVA with Bonferroni’s multiple comparisons in **e**, by one-way ANOVA with Bonferroni’s multiple comparisons in **f.**

Table S1. Proteins immunoprecipitated by GLT-1 antibody from mouse brain.
